# Supplementary material for: Genome-wide analysis of ivermectin response by Onchocerca volvulus reveals that genetic drift and soft selective sweeps contribute to loss of drug sensitivity
Source: PLoS Negl Trop Dis. 2017 Jul 26;11(7):e0005816. doi: 10.1371/journal.pntd.0005816 (PMC5546710; doi:10.1371/journal.pntd.0005816)
Supplement: S1 Text — Extended information regarding sample history and response phenotype (Section 1). Extended discussion of hard- and soft-selective sweeps (Section 2). Population assignment based on Sequenom genotyping of individual worms (Section 3). (PDF) [file pntd.0005816.s010.pdf]

S1 Text

Table of Contents

Section 1. Extended information on sample history and response phenotype 2

Table A. Criteria for phenotypic classification of *Onchocerca volvulus*. 2

Table B. Distribution of the nodule(s)[a] according to the number of fertile and the non-fertile adult *Onchocerca volvulus* worms[b] sampled in 2007 in the Nkam valley (Cameroon) prior to introduction of CDTI 3

Table C. Distribution of the nodule(s)[a] according to the number of fertile and the non-fertile adult *Onchocerca volvulus* worms[b] sampled in 2007 in the Mbam valley (Cameroon) after around 8 years of CDTI 3

Table D. Distribution of the nodule(s)[a] according to the number of fertile and the non-fertile adult *Onchocerca volvulus* worms[b] sampled in Ghana from communities exposed to 9-16 rounds of ivermectin treatment 4

Table E. Distribution of the nodule(s)[a] according to the number of fertile and the non-fertile adult *Onchocerca volvulus* worms[b] sampled from Begbomdo, Ghana that received little or no ivermectin treatment (NLT) prior to the study. 4

Fig A. Proportion of worm phenotypes by host phenotype. 5

Table F. Overview of samples selected for Pool-seq 8

Table G. Overview of samples selected for single worm genotyping 10

Table H. Sampling sites and mapping coordinates in Ghana and Cameroon. 11

Section 2. Extended discussion of hard- and soft-selective sweeps 12

Fig B. Demonstrating the consequences of hard- versus soft-selection on genetic diversity. 15

Table I. A simple multilocus quantitative trait model demonstrating that multiple genotypes can confer the same quantitative phenotype. 17

Table J. Summary of features differentiating hard and soft selection 19

Section 3. Population assignment based on Sequenom genotyping of individual worms .20

Table K. Population assignment of individual worms based on their genotype profile from 130 SNPs analysed by Sequenom genotyping 22

Fig C. Discriminant analysis of principal components analysis of genetic diversity to compare predicted versus known population assignment of individuals based on their genotype. 23

References 24

Section 1. Extended information on sample history and response phenotype

As described in the manuscript text, the samples used in the pooled genome sequencing and individual genotyping assays were acquired from sample cohorts of studies performed in Ghana [1, 2] and Cameroon [3-5]. The aim of these studies was to describe the variability in phenotypic response of *O. volvulus* to ivermectin, and the prevalence and distribution of the phenotypic classes in each study area. The criteria for classifying individual parasite samples as good responders (GR) and sub-optimal responders (SOR) to ivermectin treatment (Tx) at the host level and at the level of individual female parasites are summarized in **Table A**. These criteria were used to guide selection of worms for the genomic and genotyping analyses.

Table A. Criteria for phenotypic classification of *Onchocerca volvulus*.

**Phenotype**	**Defined at the host level**<br><br>**(microfilarial (mf) density in skin determined by skin snip)**	**Defined at parasite level**<br><br>**(intra-uterine microfilariae determined by embryogram of adult female parasites)**
Good response (GR)	Day 0 [a]: < 25 mf/mg skin<br><br>Day 80/90 [b]: 0 mf/mg skin<br><br>Day 180: < 6% of pre-Tx value	Day 80/90 [b]: Viable stretched microfilariae absent *in utero*
Sub-optimal response (SOR)	Day 80/90 [b]: > 7% of pre-Tx value	Day 80/90 [b]: Viable, stretched microfilariae present *in utero*

a. Day 0: sampling immediately before the first ivermectin treatment conducted as part of the study during which the samples were obtained
b. Day 80/90: sampling occurred on day 80 and day 90 after last ivermectin treatment conducted as part of the study in Ghana [1, 2] and Cameroon [3, 4], respectively.

To demonstrate the extent to which both adult *O. volvulus* phenotypes are present within individual hosts, we reanalysed the Ghana and Cameroon sample cohorts to identify the number of GR and SOR parasites per host (**Tables B C D and E**).

Table B. Distribution of the nodule(s)[a] according to the number of fertile and the non-fertile adult *Onchocerca volvulus* worms[b] sampled in 2007 in the Nkam valley (Cameroon) prior to introduction of CDTI

		**Number of non-fertile *O. volvulus* female worms in the nodule(s)**												
		0	***1***	***2***	***3***	***4***	***5***	***6***	***7***	***8***	***9***	***10***	***11***	***Total***
**Number of fertile *O. volvulus* female worms in the nodule(s)**	***0***		12	9	8	4	3	1					1	*38*
	1	2	5	3	2	4		2						*18*
	2	3	4	4	2	3	1							*17*
	3		1	1										*2*
	4						1							*1*
	5								1					*1*
	6													*0*
	7													*0*
	8			1										*1*
	Total	*5*	*22*	*18*	*12*	*11*	*5*	*3*	*1*	*0*	*0*	*0*	*1*	*78*[c] *(274)*[d]

a. in some cases, more than one nodule were collected at the nodulectomy site and the group of nodules was considered as one nodule.

b. fertile = with live stretched microfilariae ; non-fertile = without live stretched microfilariae

c. Total: number of hosts.

d. Total number of worms analysed in parentheses.

Table C. Distribution of the nodule(s)[a] according to the number of fertile and the non-fertile adult *Onchocerca volvulus* worms[b] sampled in 2007 in the Mbam valley (Cameroon) after around 8 years of CDTI

		**Number of non-fertile *O. volvulus* female worms in the nodule(s)**												
		0	***1***	***2***	***3***	***4***	***5***	***6***	***7***	***8***	***9***	***10***	***11***	***Total***
**Number of fertile *O. volvulus* female worms in the nodule(s)**	***0***		17	11	6	1	1	1						*37*
	1	2	2	4	4	2								*14*
	2		2	4	1	2								*9*
	3		2	1	2		1		1					*7*
	4		2			2								*4*
	5			1	1									*2*
	6													*0*
	7													*0*
	8													*0*
	Total	*2*	*25*	*21*	*14*	*7*	*2*	*1*	*1*	*0*	*0*	*0*	*0*	*73*[c] *(239)*[d]

a. in some cases, more than one nodule were collected at the nodulectomy site and the group of nodules was considered as one nodule.

b. fertile = with live stretched microfilariae ; non-fertile = without live stretched microfilariae

c. Total: number of hosts.

d. Total number of worms analysed in parentheses.

Table D. Distribution of the nodule(s)[a] according to the number of fertile and the non-fertile adult *Onchocerca volvulus* worms[b] sampled in Ghana from communities exposed to 9-16 rounds of ivermectin treatment

		Number of non-fertile *O. volvulus* female worms in the nodule(s)												
		0	***1***	***2***	***3***	***4***	***5***	***6***	***7***	***8***	***9***	***10***	***11***	***Total***
**Number of fertile *O. volvulus* female worms in the nodule(s)**	***0***		13	6	4	1	1		1					*26*
	1	5	7	3	1									*16*
	2	3	4	1	1									*9*
	3	1		1										*2*
	4													*0*
	5													*0*
	6													*0*
	7													*0*
	8													*0*
	**Total**	9	24	11	6	1	1	0	1	0	0	0	0	*53[c] (120)[d]*

a. all palpable nodules were sampled

b. fertile = with live stretched microfilariae ; non-fertile = without live stretched microfilariae

c. Total: number of hosts.

d. Total number of worms analysed in parentheses.

Table E. Distribution of the nodule(s)[a] according to the number of fertile and the non-fertile adult *Onchocerca volvulus* worms[b] sampled from Begbomdo, Ghana that received little or no ivermectin treatment (NLT) prior to the study.

		Number of non-fertile *O. volvulus* female worms in the nodule(s)												
		0	***1***	***2***	***3***	***4***	***5***	***6***	***7***	***8***	***9***	***10***	***11***	***Total***
**Number of fertile *O. volvulus* female worms in the nodule(s)**	***0***		2	3	2	1								*8*
	1			1										*1*
	2						1							*1*
	3													*0*
	4													*0*
	5													*0*
	6													*0*
	7													*0*
	8													*0*
	**Total**	0	2	4	2	1	1	0	0	0	0	0	0	*10[c] (28)[d]*

a. all palpable nodules were sampled

b. fertile = with live stretched microfilariae ; non-fertile = without live stretched microfilariae

c. Total: number of hosts.

d. Total number of worms analysed in parentheses.

Fig A shows the presence of one or both adult worm phenotypes by host phenotype, clearly demonstrating that (i) GR worms were recovered from hosts that presented with day 90 microfilarial load consistent with a host-level SOR (exclusively GR: 12.5%; n = 8; mixed GR and SOR: 15.63%, n = 10), and (ii) while worms recovered from hosts that presented with a GR response typically also had a GR phenotype (40.63%; n = 26), there was a notable proportion of GR hosts that contained only SOR worms (3.13%, n = 2), or a combination of both SOR and GR worms (17.19%; n = 11).

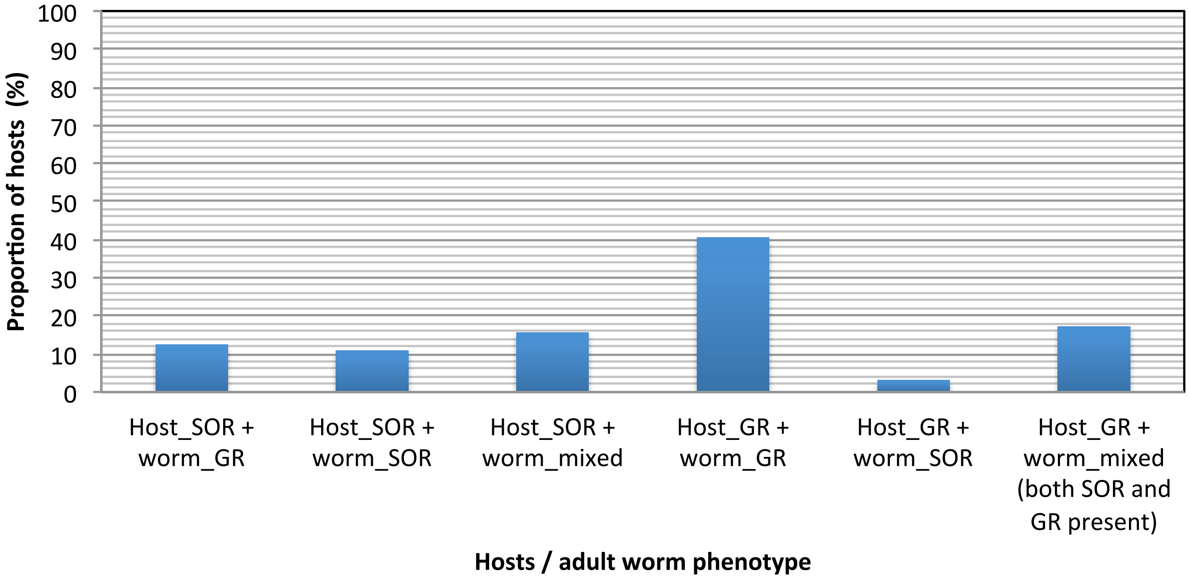

Fig A. Proportion of worm phenotypes by host phenotype.

Data represent the proportion of hosts categorised as GR or SOR based on host response (proportion of microfilariae at day 90 post treatment in the skin determined by skin snip relative to pre-treatment), that contained exclusively GR, SOR, or a mix of GR and SOR parasites.

The worms selected for Pool-seq are described in **Table F** below. The parasite samples from Ghana consisted of 3 pools made up from: (i) 10 worms from Begbomdo for the East Gonja district categorised as "drug-naïve or little treated" (NLT), from individuals having only been exposed to ivermectin treatment at days 0 and 365 prior to the time of sampling, and 90 days after ivermectin treatment on day 365; (ii) 7 "good responder" (GR) worms whose hosts lived in communities with annual CDTI and had been exposed to 11-16 annual doses of ivermectin, and (iii) 13 SOR worms from hosts living in communities with ongoing CDTI who had been exposed to 9-16 annual doses of ivermectin. The genetic data presented here (and by some of us independently; Doyle & Grant, personal communication) confirm that these 3 pools are all drawn from a single, panmictic population or transmission zone.

Similarly, the parasite samples from Cameroon used in the Pool-seq experiment consisted of 3 pools composed of (i) 40 NLT female worms from the Nkam Valley (Littoral Region; forest ecotype) which at the time of parasite collection in 2007 had not received any CDTI but which is located within 10 km of an area across the Nkam River which had at the time of sample collection received >10 annual rounds of CDTI, (ii) 22 ivermectin GR worms from the Mbam Valley (Central Region; degraded forest ecotype), and (iii) 16 SOR worms, also from the Mbam Valley which at the time of sample collection had received CDTI for 13 years. Communities from which these parasites were sampled had been exposed to MDA of ivermectin since 1994 (at least 13 years prior to sampling); however, the people from which parasites were collected were specifically exposed to between 4 to 13 ivermectin doses during a controlled clinical trial conducted between 1994 and 1997 [5]. Although samples from a number of different communities were collected within each Valley, the sampling locations were very close to each other and are certainly from a single transmission zone, at least within each valley. Hence, samples from Cameroon are described as being from the Mbam or Nkam valleys, rather than from the individual communities from which they were sampled from. The genetic data from both the pooled worm genomic analysis and from single worm genotyping presented here suggest that there was no clear distinction between samples from the Mbam and Nkam valleys. The Nkam and Mbam regions are approximately 140 km apart and separated by the Western High Plateau of Cameroon. The distance between both valleys is less than the distance between sampling sites within Ghana (~215 km). This distance is within the seasonal migratory capacity of *Simulium squamosum,* the

local vector in Cameroon [6] which can on its own cover an area of up to 20 km [7] and travel with wind assistance low hundreds of kms [6]. This upper migratory limit is consistent with seasonal migration of other members of the *S. damnosum* complex [8]. It is thus feasible that some transmission between the two regions takes place. Further work is required to confirm that blackfly cytotypes are consistent with *O. volvulus* transmission between the two sampling regions.

Parasites used in the Sequenom analysis are characterised in **Table G.** Parasites from Cameroon (n = 436) were sampled from 19 communities in the Mbam- and 7 communities in the Nkam valley regions. The samples from the Nkam valley (NKA07; n = 140, female worm tails) were characterised as NLT because they came from hosts that had been exposed to only a single ivermectin treatment 80 days prior to sampling. The samples from Mbam (MBM07; n = 112, female worm tails) came from hosts who had received between 4 and 13 treatments as part of a clinical trial undertaken between 1994 and 1997 [5] and had participated subsequently in annual CDTI in their communities; the precise number of treatments is, however, unknown. A truly ivermectin naïve group of worms from the Mbam Valley was included for comparison (MBM94; n = 184). These worms were sampled in 1994 prior to introduction of CDTI in the area [5]. In total, 184 parasites from ivermectin-naïve hosts, 225 GR parasites, and 27 SOR parasites were used. Ghanaian worms (n= 156) used for Sequenom genotyping were sampled from 6 communities (Asubende, Begbomdo, Jagbenbendo, Kyingakrom, New Longoro and Wiae), and were composed of 105 GR parasites, and 43 SOR parasites. An additional 8 parasites that had been exposed to multiple ivermectin treatment rounds but had no phenotype recorded were also included in the analyses.

Table F. Overview of samples selected for Pool-seq

Information	Ivermectin-naïve or little treated (NLT)	Good Responders (GR)	Sub-optimal responders (SOR)
**Cameroon (CAM)** [a]			
Number of worms in pool	~40 tail-less crushed female worms	22 tail-less crushed female worms	16 tail-less crushed female worms
Number of worm hosts	13	10	9
Location	Nkam Valley (Littoral region)	Mbam Valley (Central region)	Mbam Valley (Central region)
Prior ivermectin treatments of worm hosts at time of sample collection	12 worm hosts: 0<br>1 worm host: 5 annual treatments, last treatment 5 years before worm collection	4 to 13 doses of IVM between 1994 and 1997 [5]<br>Participation in annual CDTI since 1998 but records on number of treatments received not available	4 to 13 doses of IVM between 1994 and 1997 [5]<br>Participation in annual CDTI since 1998 but records on number of treatments received not available
Worm collection time	Day 0 (before ivermectin treatment)	Day 80 after last ivermectin treatment	Day 80 after last ivermectin treatment
Host-level response	**Day 80**:<br>7/13: 0% of pre-treatment mf density;<br>2/13: 3.7 and 36.8%[b] of pre-Tx mf density<br>4/13: no data<br>**Day 180:**<br>4/13: 0% of pre-Tx mf density;<br>5/13: 1.7-81.3% [c] of pre-Tx mf density<br>4/13: no data	**Day 0**: <25mf/mg skin<br>**Day 80**: 0 mf<br>**Day 180**: <6% of pre-Tx mf density	**Day 80**: >7% of pre-Tx mf density
Individual worm level response	Not determined	**Day 80**: 0 viable stretched mf	**Day 80**: viable stretched mf
**Ghana (GHA)** [a]			
Number of worms in pool	10 crushed female worms	7 crushed female worms	13 crushed female worms
Number of worm hosts	10	7	13
Locations	Begbomdo (East Gonja district)	Asubende (Atebubu district)<br>Baaya (Atebubu district)<br>Beposo (Atebubu district)	Kyingakrom (Kintampo district) (n = 4)<br>New Longoro (Kintampo district) (n = 4)<br>Jagbenbendo (East Gonja district) (n = 5)

Information	Ivermectin-naïve or little treated (NLT)	Good Responders (GR)	Sub-optimal responders (SOR)
		Hiampe (Atebubu district)<br>Senyase (Atebubu district)	
Prior annual ivermectin treatments of worm hosts at time of worm collection	2 (as part of study in which worms were collected)	11 to 16	9 to 16
Worm collection time	**Day 90** after 2nd ivermectin study Tx	**Day 90** after 2nd ivermectin study Tx	**Day 90** after 2nd ivermectin study Tx
Host-level response	**Day 90**:<br>8/10: 0 mf<br>2/10: 5.1 and 7.7% pre-Tx mf density [d]	**Day 0:** <25mf/mg skin<br>**Day 90**: 0 mf<br>**Day 180**: <6% of pre-Tx mf density	**Day 30**: with or without mf<br>**Day 90**: >7% of pre-Tx mf density
Individual worm level response	**Day 90**:<br>8/10: 0 viable stretched mf;<br>2/10: no data	**Day 90**: 0 viable stretched mf	**Day 90**: viable stretched mf

a. Details on worm collection and processing are available in the publications of the studies in which worms were collected and the phenotype characterised: Ghana [1, 2] and Cameroon [3, 4]
b. 36.8% corresponded to 0.7mf/mg at day 80
c. corresponding to 0.6 mf/mg; 1.3 mf/mg; 1.5 mf/mg; 30.6 mf/mg, 118.8 mf/mg
d. the worms that came from these 2 hosts had 0 viable stretched mf *in uteri*

Table G. Overview of samples selected for single worm genotyping

Community/ Region	Popn ID	Host prior ivermectin Tx**	Total Samples	NLT			Long term ivermectin Tx		
				GR	SOR	No pheno.	GR	SOR	No pheno
**Cameroon [a,b]**									
Nkam valley (2007)	NKA07	1 (80 days before sampling)	140 (89)	132 (84)	8 (5)		0	0	0
Mbam valley (2007)	MBM07	1 (80 days before sampling)<br>+ 4-13 doses during trial 1994-1997<br>+ unknown number during annual CDTI 1998-2006	112 (77)	0	0		93 (61)	19 (16)	0
Mbam valley (1994)	MBM94	0 (not treated)	184 (145)	0	0	184 (145)	0	0	0
**Ghana [a,b]**									
Asubende	ASU	~ 14 to 17	8 (7)	0	0	0	2 (2)	5 (5)	1
Begbomdo	BEG	2	28 (24)	25 (21)	3 (3)	0	0	0	0
Jagbenbendo	JAG	~ 9 to 12	82 (71)	0	0	0	59 (50)	19 (17)	4 (4)
Kyingakrom	KYG	~ 9 to 16	25 (22)	0	0	0	12 (11)	11 (9)	2 (2)
New Longoro	NLG	~ 10 to 16	6 (4)	0	0	0	2 (2)	4 (2)	0
Wiae	WIA	9	7 (7)	0	0	0	5 (5)	1 (1)	1 (1)
**Totals**									
Cameroon (CAM)			436 (311)	132 (84)	8 (5)	184 (145)	93 (61)	19 (16)	0 (0)
Ghana (GHA)			156 (135)	25 (21)	3 (3)	0 (0)	80 (70)	40 (34)	8 (7)
CAM plus GHA			592 (446)	157 (105)	11 (8)	184 (145)	173 (131)	59 (50)	8 (7)

a. Details on worm collection and processing are available in the publications of the studies in which worms were collected and the phenotype characterised: Ghana [1, 2], Cameroon NKA07 and MBM07 [3, 4] and Cameroon MBM94 [5].
b. The total number of parasites per community and treatment response category are presented. The actual number of parasites for which the genotype data passed filtering and were used in the analysis are in presented in parentheses.

Table H. Sampling sites and mapping coordinates in Ghana and Cameroon.

Map Reference Number [a]	Region	Community	Longitude	Latitude
**Ghana**				
1	Kintampo	Kyingakrom	8.099389	-2.115924
2	Kintampo	New Longoro	8.137722	-2.036
3	Pru	Asubende	8.019028	-0.981333
4	Pru	Baaya	8.019972	-0.995667
5	Pru	Beposo	8.007222	-0.960972
6	Pru	Ohiampe	8.007278	-1.063833
7	Pru	Senyase	8.023528	-0.990306
8	East Gonja	Begbomdo	8.523111	-0.180361
9	East Gonja	Jagbenbendo	8.294389	-0.125583
10	East Gonja	Wiae	8.32275	-0.161917
**Cameroon**				
1	Nkam	Bayon	5.059041	9.969977
2	Nkam	Ekon-Nkam	5.063759	10.015219
3	Nkam	Manjibo	5.0169807	10.0280414
4	Nkam	Mbarembeng	5.00868	10.025044
5	Nkam	Mboué	5.064808	10.003709
6	Nkam	Mounko	5.045112	10.03112
7	Nkam	Mpaka	5.015881	10.005328
8	Mbam	Balamba 1,2	4.436998	11.245767
9	Mbam	Bayomen	4.86307	11.104774
10	Mbam	Bialanguena	4.623182	11.313134
11	Mbam	Biamo	4.769267	11.205815
12	Mbam	Boalondo	4.414032	11.226201
13	Mbam	Botatango	4.415005	11.249465
14	Mbam	Boura 1	4.701287	11.294321
15	Mbam	Diodaré	4.797847	11.164902
16	Mbam	Gah	4.807858	11.14686
17	Mbam	Kalong	4.847731	11.121539
18	Mbam	Kon	4.834719	11.056581
19	Mbam	Lablé	4.752668	11.250728
20	Mbam	Lakpang	4.793297	11.172188
21	Mbam	Ngomo	4.771423	11.284028
22	Mbam	Nyamanga	4.581044	11.334097
23	Mbam	Nyamsong	4.758291	11.259373
24	Mbam	Nyatsotta	4.777883	11.290298
25	Mbam	Yambetta	4.81672	10.980783
26	Mbam	Yébékolo	4.530996	11.322509

a. Map reference numbers refer to locations shown in **Fig 1** in the main manuscript**.**

Section 2. Extended discussion of hard- and soft-selective sweeps

Genetic change in a population (**Fig B (i)**) in response to a specific selection pressure (such as anthelmintic treatment) is generally conceptualised as proceeding by one of two genetic mechanisms [9, 10].

In the first, generally termed *hard selection*, a rare mutation that has arisen around the time of the first application of the selective pressure (in this case, ivermectin), and which is sufficient to confer increased survival/fecundity following anthelmintic exposure, becomes rapidly fixed (i.e. replaces all other alleles present at the time of start of selection pressure) in the population (**Fig B (ii)** and **(iii)**). The rarity, recent origin and rapid fixation of the allele in the population means that the genomic region surrounding the allele will "hitchhike" into the resistant population along with the resistance allele, and thus also become fixed. Since this is likely to occur over only a few generations, there is little or no recombination around the allele under selection and thus there is a large region of the genome surrounding the resistance allele that is shared between all members of the resistant population i.e., is in strong linkage disequilibrium (LD) with the resistance allele. Thus, a genome-wide association study (GWAS) detects selection of resistance conferring alleles at the resistance locus as regions of strong LD or increased genetic differentiation (e.g., by Wright's F_{ST} statistic) surrounding causative alleles between susceptible and resistant organisms. That strong signal of genetic differentiation, with the same allele in the same "hitchhiking" surrounding genomic environment, will be present in all survivors of treatment in that population and their progeny provided they contain the resistance conferring allele.

Depending on the mechanism of the resistance, comparison between populations in which resistance has been selected independently may show hard selection of the same or different allele of the same locus, or different alleles at a different locus if more than one resistance mechanism is possible. The defining feature, therefore, of "textbook" hard selection is that in any given population, one allele at a single locus goes rapidly to fixation, accompanied by fixation of the genomic region surrounding the selected allele. This leads to strong local LD and reduced local genetic variation, particularly in the genomic "neighbourhood" of the selected locus but also more generally throughout the genome.

An illustration of hard selection in the human population is the independent selection in Europe and Asia for persistence of lactase expression in adults [11]. Lactase is the enzyme that catalyses the first step in lactose metabolism and, in most mammals, expression of lactase is much reduced or absent in adults resulting in lactose intolerance. In humans, the evolution of lactase expression in adults accompanied the domestication of dairy animals and inclusion of dairy products in the human diet. The same changes in lactase expression evolved independently in all human populations that utilise dairy products, leaving strong signatures of hard selection in the genomic region surrounding the lactase gene in the genomes of those populations.

In the second form of selection, generally termed *soft selection*, the trait under selection (anthelmintic resistance) is determined by small contributions from alleles at many loci i.e., resistance is a polygenic, quantitative trait (QT) (**Fig B (iv)**). A further important defining feature of a QT is that the same phenotype (or trait value) can be achieved by the additive contributions of different alleles at different loci (quantitative trait loci, QTL), such that two individuals within a population may show the same trait value (degree of anthelmintic resistance, phenotype) but have different genotypes (see **Table I** for a simple model example of a 4 genotype / phenotype interaction demonstrated using a punnett square). The same phenomenon in which many genotypes give rise to the same phenotype can occur between two populations in which selection has occurred independently. Consequently, two populations may show different signals of genetic differentiation (i.e. different genotypes associated with resistance) due to the combined additive effects of different loci in each population, even though they are phenotypically equivalent. Furthermore, because many loci may each make a small contribution, the genetic “signature” of selection is divided amongst those loci (which may differ between individuals within a population and between populations); the genetic signal of selection for any one locus may be weak (**Fig B (v)**), and therefore, fixation of quantitative trait loci is unusual.

Given that many alleles at many loci are under selection for a QT and that mutation creates new alleles slowly, most or all of the alleles that contribute to a QT are present in the “standing genetic variation” (existing genetic variation) that is a feature of all populations (i.e, **Fig B (i))**. Furthermore, some of these alleles may have been present in the population for many generations, so that recombination has had sufficient time to re-shuffle these

alleles onto many different genetic backgrounds or haplotypes before selection is imposed. This feature of QTL means that the alleles under selection generally occur on many different haplotypes, or genetic backgrounds (**Fig B (iv)**), so there is usually only weak, and variable, LD between the allele under selection and its immediate genomic neighbourhood. The lack of LD following selection contributes to a further weakening of the post-selection genetic signature, and ensures that genetic variation is maintained in the post-selection (i.e., anthelmintic resistant) population (**Fig B (v)**). Consequently, and in contrast to hard selection, the selected population maintains similar levels of genetic variation to the pre-selection, drug-naïve, population.

The terms hard and soft selection refer, therefore, to the genetic basis of the trait under selection. The contrasting outcomes of hard and soft selection are the products of the different history of the selected alleles in hard and soft selection, the post-selection genetic similarity/difference between individuals and populations, and the extent to which LD creates a signal of reduced genetic diversity surrounding the selected allele (see **Table J** for a summary of characteristics that differentiate hard and soft selection). The terms hard and soft do not refer at all to the strength of selection nor to the speed of the selection response. Phenotypic change in populations due to soft selection can be just as rapid and just as profound as for hard selection.

From a human evolutionary perspective, there are many recently characterised examples of the evolution of QTs by soft selection. One is the evolution of paler skin colour in high latitude populations [9]. This trait has evolved independently in European and Asian populations, requires contributions from at least several and likely more genes, and may have left different signatures of selection in different populations.

Fig B. Demonstrating the consequences of hard- versus soft-selection on genetic diversity.

Prior to the application of a selection pressure, a population (depicted by 10 “genomes” [horizontal lines]) will typically contain a given amount of standing genetic variation (black filled circles) (i). On the application of a selection pressure in which a single, rare genetic variant provides a significant fitness advantage, a *hard selective sweep* (ii; red star) may take place, whereby the initially rare variant becomes highly prevalent and may become fixed in the population while the selection pressure continues to be applied (iii). The consequences of this are that the causative variant, and all variants physically linked to that variant (via linkage disequilibrium) increase in frequency, and in turn, reduce or remove all other genetic variation present in the population. (iv) Alternatively, if there are many variants in a population that provide small or moderate fitness advantages in the presence of a selective pressure, i.e., quantitative trait loci (QTLs; different shades of orange polygons), a *soft selective sweep* may occur, in which some of those QTL may increase in frequency, but are unlikely to become fixed in the population. As these beneficial variants exist in different genetic backgrounds prior to selection, these additional variants will be retained and hence, overall genetic variation of the population will be somewhat maintained, depending on the number of QTLs and strength of selection (v).

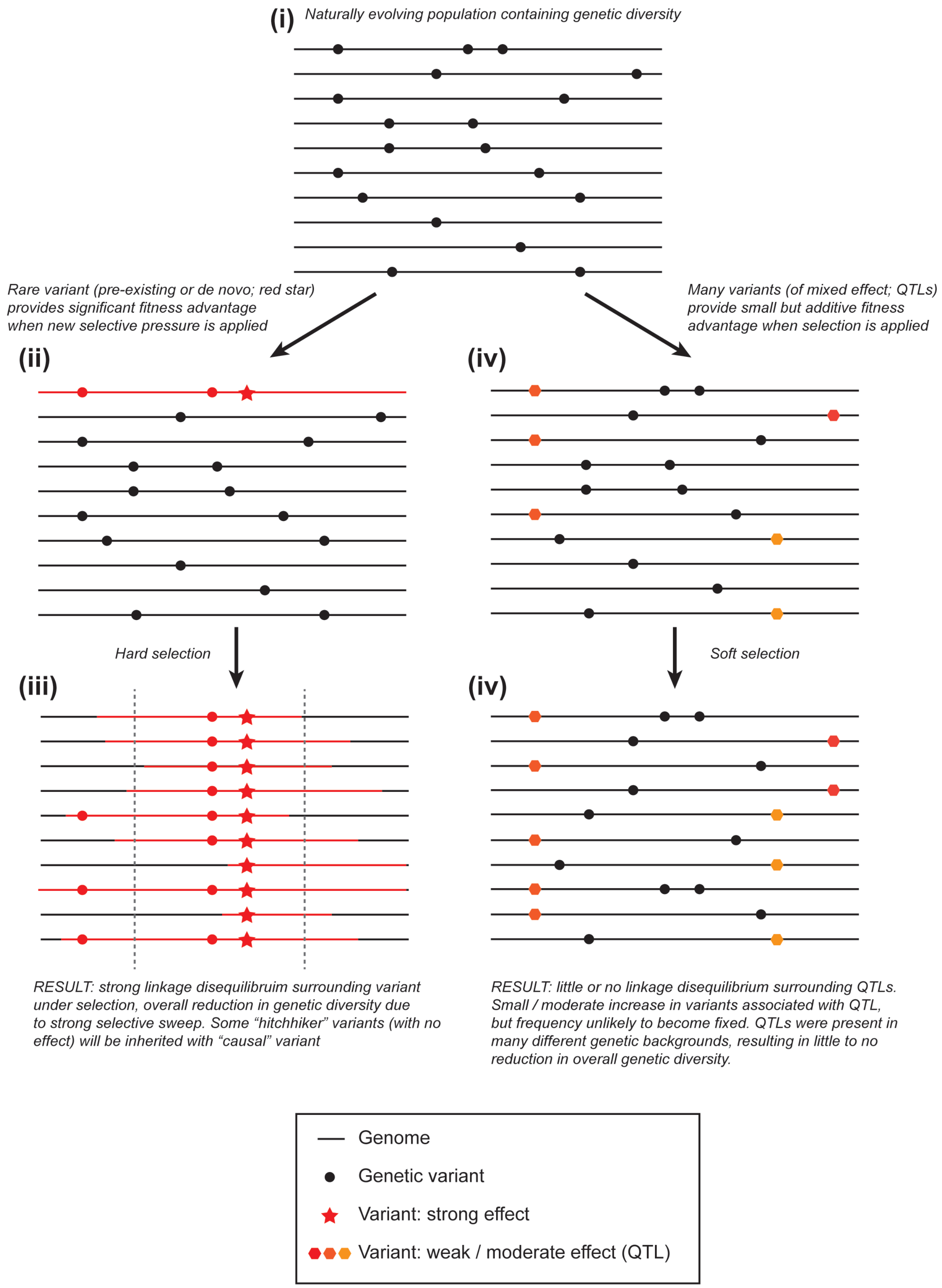
(i) Naturally evolving population containing genetic diversity
Rare variant (pre-existing or de novo; red star) provides significant fitness advantage when new selective pressure is applied
Many variants (of mixed effect; QTLs) provide small but additive fitness advantage when selection is applied
(ii)
(iv)
Hard selection
Soft selection
(iii)
(iv)
RESULT: strong linkage disequilibruim surrounding variant under selection, overall reduction in genetic diversity due to strong selective sweep. Some “hitchhiker” variants (with no effect) will be inherited with “causal” variant
RESULT: little or no linkage disequilibrium surrounding QTLs. Small / moderate increase in variants associated with QTL, but frequency unlikely to become fixed. QTLs were present in many different genetic backgrounds, resulting in little to no reduction in overall genetic diversity.
Genome
Genetic variant
Variant: strong effect
Variant: weak / moderate effect (QTL)

Table I. A simple multilocus quantitative trait model demonstrating that multiple genotypes can confer the same quantitative phenotype.

	ABCD	***Abcd***	***aBCD***	***ABcd***	***abCD***	***ABCd***	***abcD***	***AbcD***	***aBCd***	***ABcD***	***abCd***	***AbCD***	***aBcd***	***AbCd***	***aBcD***	***abcd***
ABCD	ABCD<br>ABCD	ABCD<br>Abcd	ABCD<br>aBCD	ABCD<br>ABcd	ABCD<br>abCD	ABCD<br>ABCd	ABCD<br>abcD	ABCD<br>AbcD	ABCD<br>aBCd	ABCD<br>ABcD	ABCD<br>abCd	ABCD<br>AbCD	ABCD<br>aBcd	ABCD<br>AbCd	ABCD<br>aBcD	ABCD<br>abcd
Abcd	Abcd<br>ABCD	Abcd<br>Abcd	Abcd<br>aBCD	Abcd<br>ABcd	Abcd<br>abCD	Abcd<br>ABCd	Abcd<br>abcD	Abcd<br>AbcD	Abcd<br>aBCd	Abcd<br>ABcD	Abcd<br>abCd	Abcd<br>AbCD	Abcd<br>aBcd	Abcd<br>AbCd	Abcd<br>aBcD	Abcd<br>abcd
aBCD	aBCD<br>ABCD	aBCD<br>Abcd	aBCD<br>aBCD	aBCD<br>ABcd	aBCD<br>abCD	aBCD<br>ABCd	aBCD<br>abcD	aBCD<br>AbcD	aBCD<br>aBCd	aBCD<br>ABcD	aBCD<br>abCd	aBCD<br>AbCD	aBCD<br>aBcd	aBCD<br>AbCd	aBCD<br>aBcD	aBCD<br>abcd
ABcd	ABcd<br>ABCD	ABcd<br>Abcd	ABcd<br>aBCD	ABcd<br>ABcd	ABcd<br>abCD	ABcd<br>ABCd	ABcd<br>abcD	ABcd<br>AbcD	ABcd<br>aBCd	ABcd<br>ABcD	ABcd<br>abCd	ABcd<br>AbCD	ABcd<br>aBcd	ABcd<br>AbCd	ABcd<br>aBcD	ABcd<br>abcd
abCD	abCD<br>ABCD	abCD<br>Abcd	abCD<br>aBCD	abCD<br>ABcd	abCD<br>abCD	abCD<br>ABCd	abCD<br>abcD	abCD<br>AbcD	abCD<br>aBCd	abCD<br>ABcD	abCD<br>abCd	abCD<br>AbCD	abCD<br>aBcd	abCD<br>AbCd	abCD<br>aBcD	abCD<br>abcd
ABCd	ABCd<br>ABCD	ABCd<br>Abcd	ABCd<br>aBCD	ABCd<br>ABcd	ABCd<br>abCD	ABCd<br>ABCd	ABCd<br>abcD	ABCd<br>AbcD	ABCd<br>aBCd	ABCd<br>ABcD	ABCd<br>abCd	ABCd<br>AbCD	ABCd<br>aBcd	ABCd<br>AbCd	ABCd<br>aBcD	ABCd<br>abcd
abcD	abcD<br>ABCD	abcD<br>Abcd	abcD<br>aBCD	abcD<br>ABcd	abcD<br>abCD	abcD<br>ABCd	abcD<br>abcD	abcD<br>AbcD	abcD<br>aBCd	abcD<br>ABcD	abcD<br>abCd	abcD<br>AbCD	abcD<br>aBcd	abcD<br>AbCd	abcD<br>aBcD	abcD<br>abcd
AbcD	AbcD<br>ABCD	AbcD<br>Abcd	AbcD<br>aBCD	AbcD<br>ABcd	AbcD<br>abCD	AbcD<br>ABCd	AbcD<br>abcD	AbcD<br>AbcD	AbcD<br>aBCd	AbcD<br>ABcD	AbcD<br>abCd	AbcD<br>AbCD	AbcD<br>aBcd	AbcD<br>AbCd	AbcD<br>aBcD	AbcD<br>abcd
aBCd	aBCd<br>ABCD	aBCd<br>Abcd	aBCd<br>aBCD	aBCd<br>ABcd	aBCd<br>abCD	aBCd<br>ABCd	aBCd<br>abcD	aBCd<br>AbcD	aBCd<br>aBCd	aBCd<br>ABcD	aBCd<br>abCd	aBCd<br>AbCD	aBCd<br>aBcd	aBCd<br>AbCd	aBCd<br>aBcD	aBCd<br>abcd
ABcD	ABcD<br>ABCD	ABcD<br>Abcd	ABcD<br>aBCD	ABcD<br>ABcd	ABcD<br>abCD	ABcD<br>ABCd	ABcD<br>abcD	ABcD<br>AbcD	ABcD<br>aBCd	ABcD<br>ABcD	ABcD<br>abCd	ABcD<br>AbCD	ABcD<br>aBcd	ABcD<br>AbCd	ABcD<br>aBcD	ABcD<br>abcd
abCd	abCd<br>ABCD	abCd<br>Abcd	abCd<br>aBCD	abCd<br>ABcd	abCd<br>abCD	abCd<br>ABCd	abCd<br>abcD	abCd<br>AbcD	abCd<br>aBCd	abCd<br>ABcD	abCd<br>abCd	abCd<br>AbCD	abCd<br>aBcd	abCd<br>AbCd	abCd<br>aBcD	abCd<br>abcd
AbCD	AbCD<br>ABCD	AbCD<br>Abcd	AbCD<br>aBCD	AbCD<br>ABcd	AbCD<br>abCD	AbCD<br>ABCd	AbCD<br>abcD	AbCD<br>AbcD	AbCD<br>aBCd	AbCD<br>ABcD	AbCD<br>abCd	AbCD<br>AbCD	AbCD<br>aBcd	AbCD<br>AbCd	AbCD<br>aBcD	AbCD<br>abcd
aBcd	aBcd<br>ABCD	aBcd<br>Abcd	aBcd<br>aBCD	aBcd<br>ABcd	aBcd<br>abCD	aBcd<br>ABCd	aBcd<br>abcD	aBcd<br>AbcD	aBcd<br>aBCd	aBcd<br>ABcD	aBcd<br>abCd	aBcd<br>AbCD	aBcd<br>aBcd	aBcd<br>AbCd	aBcd<br>aBcD	aBcd<br>abcd
AbCd	AbCd<br>ABCD	AbCd<br>Abcd	AbCd<br>aBCD	AbCd<br>ABcd	AbCd<br>abCD	AbCd<br>ABCd	AbCd<br>abcD	AbCd<br>AbcD	AbCd<br>aBCd	AbCd<br>ABcD	AbCd<br>abCd	AbCd<br>AbCD	AbCd<br>aBcd	AbCd<br>AbCd	AbCd<br>aBcD	AbCd<br>abcd
aBcD	aBcD<br>ABCD	aBcD<br>Abcd	aBcD<br>aBCD	aBcD<br>ABcd	aBcD<br>abCD	aBcD<br>ABCd	aBcD<br>abcD	aBcD<br>AbcD	aBcD<br>aBCd	aBcD<br>ABcD	aBcD<br>abCd	aBcD<br>AbCD	aBcD<br>aBcd	aBcD<br>AbCd	aBcD<br>aBcD	aBcD<br>abcd
abcd	abcd<br>ABCD	abcd<br>Abcd	abcd<br>aBCD	abcd<br>ABcd	abcd<br>abCD	abcd<br>ABCd	abcd<br>abcD	abcd<br>AbcD	abcd<br>aBCd	abcd<br>ABcD	abcd<br>abCd	abcd<br>AbCD	abcd<br>aBcd	abcd<br>AbCd	abcd<br>aBcD	abcd<br>abcd
**Alleles (#)**		8	7	6	5	4	3	2	1	0						
**Phenotype (early fecundity; weeks)**		8	7	6	5	4	3	2	1	0						

In **Table I**, we present a hypothetical example of multigenic trait in which 4 bi-allelic loci contribute to a phenotype. In this example, we have defined the trait as the number of weeks to presence of stretched microfilariae (mf) *in utero*, and that the alleles designated by uppercase letters (*A, B, C, D*) are "SOR" alleles that can each individually advance the appearance of stretched mf *in utero* by one week. For example, the genotype ABCD/ABCD confers a phenotype of 8-week earlier recovery of fecundity compared to a fully susceptible *abcd/abcd* genotype. Any combination of 6 "SOR" alleles confers a 6-week advance, any combination of 4 SOR alleles a 4 week advance etc. The lowercase alleles *a, b, c, d* (GR alleles) do not confer any reduction in the time to the appearance of stretched mf *in utero* and so have a trait value of zero. Although this is an over simplified example, the punnett square in **Table I** does make clear that there are several different genotypes that can confer the same trait value (by adding all the uppercase alleles in the genotype), so that there are multiple genotypes that specify each phenotypic category (the colour coding).

Table J. Summary of features differentiating hard and soft selection

Hard selection	Soft selection
Alleles under selection arise at around the same time as selection is first imposed.	Alleles under selection are part of the existing, standing genetic variation of the population and may have been present for many generations.
Single or very few loci involved; simple mendelian trait.	Many loci involved; polygenic quantitative trait.
Each allele makes a large contribution to the phenotype.	Each allele makes a small, often additive, contribution to the trait.
Selection acts on a single (or very few) rare, new or recent alleles	Selection acts on many alleles scattered throughout the genome. Alleles vary in age but pre-date the imposition of selection.
Genetic hitchhiking between the allele under selection and its immediate genomic neighbours gives rise to strong linkage disequilibrium and thus strong signals of selection in a genome-wide association study (GWAS).	Because the many alleles under selection were present before selection, prior recombination between these alleles and their genomic neighbours has occurred and post-selection linkage disequilibrium is weak, giving rise to weaker signals of selection.
The genetic outcome is the same or very similar in independent populations exposed to the same selection.	The genetic outcome can be very different in independent populations exposed to the same selection, even when the phenotypic outcomes are the same.
The allele under selection usually goes to fixation i.e. replaces all other alleles present at the time of selection.	Alleles under selection rarely go to fixation because many different alleles at different loci make similar contributions to the phenotype, so that selection on any given allele is relatively weak.
The extent of phenotypic change can be very large over few generations.	The extent of phenotypic change can be very large over few generations.

Section 3. Population assignment based on Sequenom genotyping of individual worms

The data presented in **Fig 4** of the main manuscript demonstrate that genetic diversity among individual worm samples is driven primarily by large-scale geographic differences (**Fig 4A**: Cameroon worms are clearly different from Ghana worms). Some within-country structure is present, but there is a significant temporal effect in the Cameroon populations, given that the MBM94 and MBM07/NKA07 populations were sampled approximately 13 years apart (**Fig 4B**). Considering that ivermectin-treatment occurred during those 13 years, it is likely that this genetic change is in-part due to ivermectin treatment. However, the distinction between ivermectin-mediated genetic change due to restriction of transmission and increased stochastic processes such as genetic drift, and ivermectin-mediated genetic change due to selection of SOR phenotypes, is critically important. We believe that the data support the former. Worms which have been phenotypically classified as SOR are genetically diverse, suggesting that the genetic determinations of the SOR phenotype are present in different genetic backgrounds, and that they do not share common ancestry; extensive shared ancestry would be expected if genetic change was occurring primarily due to strong selection of SOR phenotypes. We do not have genotype/phenotype data for the Nkam population sampled in 1994 (i.e., time-matched to the MBM94 population, or phenotype data for the MBM94 population), and therefore, we cannot directly compare or estimate the increase in the proportion of SOR phenotypes in a single population over time. However, the fact that the SOR frequency is only marginally different between the single-treated Nkam population (~5%) and the upper limit of 13-18 ivermectin treatments of the Mbam population (~16%) at the time of sampling in 2007, suggests that although SOR phenotypes may be increasing in response to treatment, there is a readily detectable proportion of SOR phenotypes already in these populations prior to- or at first treatment with ivermectin. Therefore, the significant genetic change between MBM94 and MBM07 is unlikely to be driven by the (assumed) small increase in SOR phenotypes in this population.

A complementary approach to the MDS analysis presented in **Fig 4** of the main manuscript is to attempt to assign a given individual to its “correct” or “known” population, based on its genetic profile alone. This approach aims to genetically predict to where an individual belongs, and given that we know where an individual does come from, we can determine how informative a genotype may be based on the likelihood or probability of the

assignment being correct. This type of analysis will be critically important in the development of genetic markers to predict SOR, as it will provide an estimate of the "informativeness" of a marker, and can be used to determine the number of markers required (based on their "informativeness") to achieve a desired predictive power for discriminating SOR from GR phenotypes based on the genotype alone. We have applied such an approach using discriminant analysis of principal components (DAPC) and population assignment to determine the predictive capability of the single worm genotypes to assign worms to the three levels of stratification presented in **Fig 4** in the main manuscript, i.e., country (**Fig 4A**), community / region (**Fig 4B**), and response type (**Fig 4C**). These results are presented in **Table K** and **Fig C**.

Consistent with clear geographic distinction between Ghana and Cameroon samples in **Fig 4A**, approximately 99.6% of the samples were correctly assigned to their country of origin based on genotype alone. When the samples were assigned based on community or region, a greater assignment rate was achieved for samples from Cameroon (which did show some structure in **Fig 4B**) compared to samples from Ghana (which did not), and particularly for the MBM94 samples with a correct assignment of 92.4%. The total assignment proportion was relatively low (66.9%), which is driven largely by the complete lack of assignment in a number of Ghanaian communities (ASU, BEG, KYG, NLG). Surprisingly, worms from the JAG community in Ghana were discriminated / assigned well (97.2%), despite not being obviously differentially clustered in **Fig 4B**. Assignment based on treatment and treatment response demonstrated a striking difference in assignment success between naïve/GR and SOR individuals: (i) naïve individuals were assigned well (93.8%), however, they were all derived from the MBM94 population which was distinct in the analysis of community/regions, and demonstrates clearly the challenge of disconnecting a treatment condition from the underlying population structure from which those samples were collected from; (ii) the GR samples from both Cameroon (95.2%) and Ghana (95.6%) also were well assigned, and may reflect that they were at a high frequency in both countries; (iii) in both countries, the assignment of SOR individuals to the SOR groups was very poor, with no Cameroon samples and only 8.1% of Ghana samples correctly assigned to the SOR groups.

These results further support the genetic relatedness analysis presented in **Fig 4**, suggesting that underlying geographic and temporal differences between the some of the sample sites account for more of the genetic variation than that driven by SOR samples, and that the significant genetic change between the Mbam samples from 1994 and 2007 is likely due to ivermectin-mediated population dynamic changes (decreased transmission, increased bottlenecking, increased genetic drift effects) that would be expected under mass drug administration, and unlikely to be caused by genetic change due to SOR.

Table K. Population assignment of individual worms based on their genotype profile from 130 SNPs analysed by Sequenom genotyping

**Proportion of worms correctly assigned**									
To country of origin									
Total	*Cameroon*		*Ghana*						
0.996	0.997		0.993						
To region/community of origin									
Total	*NKA 07*	*MBM 94*	*MBM 07*	*ASU*	*BEG*	*JAG*	*KYG*	*NLG*	*WIA*
0.669	0.697	0.924	0.403	0.000	0.000	0.972	0.000	0.000	0.286
To response phenotype									
Total	*Cam: GR*	*Cam: naïve*	*Cam: SOR*	*Gha: GR*	*Gha: SOR*	*Gha: no phenotype*			
0.816	0.952	0.938	0.000	0.956	0.081	0.000			

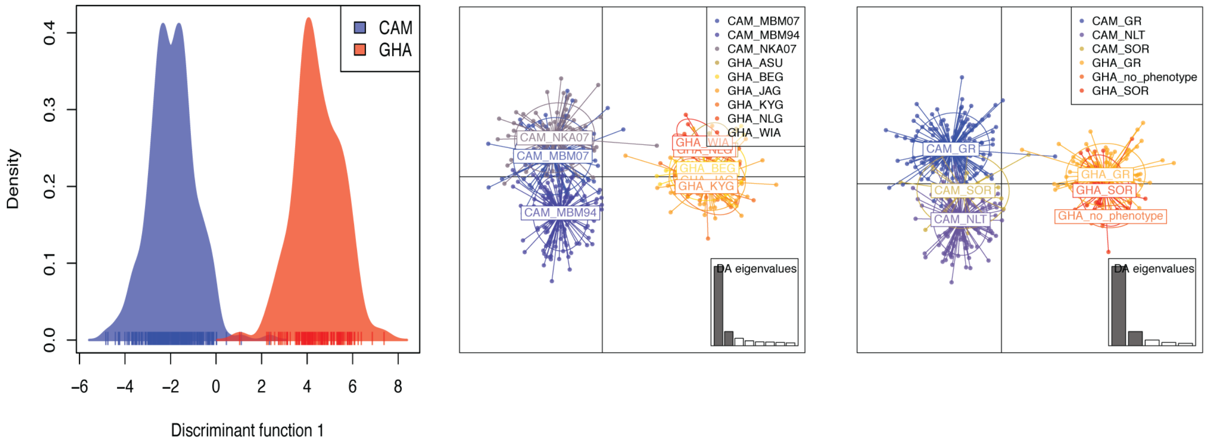

Fig C. Discriminant analysis of principal components analysis of genetic diversity to compare predicted versus known population assignment of individuals based on their genotype.

Data are analysed by country (A), community or region (B), and ivermectin treatment and response (C). Data are the same as presented in **Fig 4** of the main manuscript, which includes 446 samples genotyped at 130 loci by Sequenom genotyping.

References

1. Osei-Atweneboana MY, Awadzi K, Attah SK, Boakye DA, Gyapong JO, Prichard RK. Phenotypic evidence of emerging ivermectin resistance in *Onchocerca volvulus*. PLoS Negl Trop Dis. 2011;5(3):e998.

2. Osei-Atweneboana MY, Eng JK, Boakye DA, Gyapong JO, Prichard RK. Prevalence and intensity of *Onchocerca volvulus* infection and efficacy of ivermectin in endemic communities in Ghana: a two-phase epidemiological study. Lancet. 2007;369(9578):2021-9.

3. Nana-Djeunga HC, Bourguinat C, Pion SDS, Bopda J, Kengne-Ouafo JA, Njiokou F, et al. Reproductive status of *Onchocerca volvulus* after ivermectin treatment in an ivermectin-naive and a frequently treated population from Cameroon. PLoS Negl Trop Dis. 2014;8(4):e2824.

4. Pion SDS, Nana-Djeunga HC, Kamgno J, Tendongfor N, Wanji S, Njiokou F, et al. Dynamics of *Onchocerca volvulus* microfilarial densities after ivermectin treatment in an ivermectin-naive and a multiply treated population from Cameroon. PLoS Negl Trop Dis. 2013;7(2):e2084.

5. Gardon J, Boussinesq M, Kamgno J, Gardon-Wendel N, Demanga N, Duke BO. Effects of standard and high doses of ivermectin on adult worms of *Onchocerca volvulus*: a randomised controlled trial. Lancet. 2002;360(9328):203-10.

6. Traore-Lamizana M, Lemasson J. Participation à une étude de faisabilité d'une campagne de lutte contre l'onchocercose dans la région du bassin du Logone: répartition des espèces du complexe *Simulium damnosum* dans la zone camerounaise du projet. Cahiers-ORSTOM Entomologie médicale et parasitologie. 1988;25(3-4):171-86.

7. Thompson BH. Studies on the flight range and dispersal of Simulium damnosum (Diptera: Simuliidae) in the rain-forest of Cameroon. Ann Trop Med Parasitol. 1976;70(3):343-54.

8. Garms R, Walsh JF, Davies JB. Studies on the reinvasion of the Onchocerciasis Control Programme in the Volta River Basin by Simulium damnosum s.I. with emphasis on the south-western areas. Tropenmed Parasitol. 1979;30(3):345-62.

9. Messer PW, Petrov DA. Population genomics of rapid adaptation by soft selective sweeps. Trends in Ecology & Evolution. 2013;28(11):659-69.

10. Pritchard JK, Pickrell JK, Coop G. The genetics of human adaptation: hard sweeps, soft sweeps, and polygenic adaptation. Curr Biol. 2010;20(4):R208-15.

11. Tishkoff SA, Reed FA, Ranciaro A, Voight BF, Babbitt CC, Silverman JS, et al. Convergent adaptation of human lactase persistence in Africa and Europe. Nat Genet. 2007;39(1):31-40.
